# Supplementary material for: Cytokinesis in vertebrate cells initiates by contraction of an equatorial actomyosin network composed of randomly oriented filaments
Source: eLife. 2017 Nov 6;6:e30867. doi: 10.7554/eLife.30867 (PMC5673306; doi:10.7554/eLife.30867)
Supplement: Transparent reporting form [file elife-30867-transrepform.docx]

***eLife’s* transparent reporting form**

We encourage authors to provide detailed information *within their submission* to facilitate the interpretation and replication of experiments. If you have any questions, please contact us: [editorial@elifesciences.org](mailto:editorial@elifesciences.org).

**Sample-size estimation**

- You should state whether an appropriate sample size was computed when the study was being designed
- You should state the statistical method of sample size computation and any required assumptions
- If no explicit power analysis was used, you should describe how you decided what sample (replicate) size (number) to use

Please outline where this information can be found within the submission (e.g., page numbers or figure legends), or explain why this information doesn’t apply to your submission:

We chose sample sizes according to widely accepted standards in the cell biology field, which take into account the experimental effort required to generate the respective data. Statistical methods of sample size computation were not used.

**Replicates**

- You should report how often each experiment was performed
- You should include a definition of biological versus technical replication
- The data obtained should be provided and sufficient information should be provided to indicate the number of independent biological and/or technical replicates
- If you encountered any outliers, you should describe how these were handled
- Criteria for exclusion/inclusion of data should be clearly stated
- High-throughput sequence data should be uploaded before submission, with a private link for reviewers provided (these are available from both GEO and ArrayExpress)

Please outline where this information can be found within the submission (e.g., page numbers or figure legends), or explain why this information doesn’t apply to your submission:

Number of biological replicates is indicated in the main text and figure legends for each experiment. Every experiment was performed at least three times in independent biological replicates. No data points were excluded for analysis.

**Statistical reporting**

- Statistical analysis methods should be described and justified
- Raw data should be presented in figures whenever informative to do so (typically when N per group is less than 10)
- For each experiment, you should identify the statistical tests used, exact values of N, definitions of center, methods of multiple test correction, and dispersion and precision measures (e.g., mean, median, SD, SEM, confidence intervals; and, for the major substantive results, a measure of effect size (e.g., Pearson's r, Cohen's d)
- Report exact p-values wherever possible alongside the summary statistics and 95% confidence intervals. These should be reported for all key questions and not only when the p-value is less than 0.05.

For statistical analysis of Fig. 1G a Kolmogorov Smirnov test was used, for Fig4G-I a single-tailed Mann-Whitney U tests were used, and for Fig. 2 Supplement 1J a two-tailed Mann-Whitney U test was used. These tests are non parametric and do not require normal distributed data.

For statistical analysis of Fig. 2E and F two tailed t-tests were used. Values from all timepoints are normal distributed according to Kolmogorov Smirnov tests.

Please outline where this information can be found within the submission (e.g., page numbers or figure legends), or explain why this information doesn’t apply to your submission:

n.s. = not significant

**Figure 1G**

0-100

Equator (median=0.483, n=26) – pole (median=0.4178, n=26); p=0.0887; n.s.

101-150

Equator (median=0.4881, n=35) – pole (median=0.468, n=35); p=0.1975; n.s.

151-200

Equator (median=0.3894, n=36) – pole (median=0.5466, n=36); p=0.0000008305;

201-300

Pole (median=0.3187, n=39) – pole (median=0.5692, n=39); p=0.000000000000046

**Figure 2 E**

-90 s to -30 (median= -0.09133, n=46) vs. 0 s (median = -0.09444, n= 18); p=0.8390; n.s.

-90 s to -30 (median= -0.09133, n=46) vs. 30 s (median= -0.0885, n= 18); p=0.9672; n.s.

-90 s to -30(median= -0.09133, n=46) vs. 60 s (median= -0.08571, n=18); p=0.2851; n.s.

-90 s to -30(median= -0.09133, n=46) vs. 90 s (median = -0.05796, n=18); p=0.0004;

**Figure 2F**

-90 s to -30 (median= 1.001, n=46) vs. 0 s (median = 1.014, n= 18); p=0.5221; n.s.

-90 s to -30 (median= 1.001, n=46) vs. 30 s (median= 1.022, n= 18); p=0.2120; n.s.

-90 s to -30 (median= 1.001, n=46) vs. 60 s (median= 1.011, n=18); p=0.2100; n.s.

-90 s to -30 (median= 1.001, n=46) vs. 90 s (median = 0.9934, n=18); p=0.8555; n.s.

-90 s to -30 (median= 1.001, n=46) vs. 120 s (median = 0.9665, n=18); p=0.0014

**Figure 2 Supplement 1J**

Metaphase (mean=213.2, median=194.8, n=48) – Anaphase (mean=176.3, median=178.8, n=50); p=0.2993; n.s.

Metaphase (mean=213.2, median=194.8, n=48) – Interphase (mean=303.4, median=290.9, n=71); p=0.0026

**Figure 4G**

Metaphase (median=0.1913, n=20) – pre-ingression (median=0.1201, n=10); p = 0.1009; n.s.

Metaphase (median=0.1913, n=20) – early ingression (median=0.4396, n=15); p = 0.0035

Metaphase (median=0.1913, n=20) – late ingression (median=0.893, n=14); p =3.45E-06

**Figure 4H**

Metaphase (median=0.1707, n=10) - pre-ingression (median=0.1275, n=9); p = 0.4524; n.s.

Metaphase (median=0.1707, n=10) – early ingression (median=0.2253, n=15); p = 0.0981; n.s.

Metaphase (median=0.1707, n=10) – late ingression (median=0.3339,n=4); p = 0.0939; n.s

**Figure 4I**

Metaphase pre cut (median=-2.494e-017, n=8) – metaphase post cut (median=-0.05886, n=8); p = 0.2527; n.s.

Early ingression pre cut (median=0.2565, n=12) – early ingression post cut (median=0.2323, n=12); p = 0.3353; n.s.

Late ingression pre cut (median=0.4603, n=8) – late ingression post cut (median=0.3573, n=8); p = 0.0974; n.s.

(For large datasets, or papers with a very large number of statistical tests, you may upload a single table file with tests, Ns, etc., with reference to page numbers in the manuscript.)

**Additional data files (“source data”)**

- We encourage you to upload relevant additional data files, such as numerical data that are represented as a graph in a figure, or as a summary table
- Where provided, these should be in the most useful format, and they can be uploaded as “Source data” files linked to a main figure or table
- Include model definition files including the full list of parameters used
- Include code used for data analysis (e.g., R, MatLab)
- Avoid stating that data files are “available upon request”

Please indicate the figures or tables for which source data files have been provided:

We provided a set of custom-developed MatLab scripts that segment cells and perform measurements on cortical intensities based on the segmented regions and measure various cell parameters like chromatin-chromatin distance and cleavage furrow diameter.
